# Preparation, Statistical Optimization and Characterization of Propolis-Loaded Solid Lipid Nanoparticles Using Box-Behnken Design

**DOI:** 10.34172/apb.2021.043

**Published:** 2020-06-20

**Authors:** Sahar Taherzadeh, Atefeh Naeimifar, Ehsan Mehrabani Yeganeh, Zahra Esmaili, Reza Mahjoub, Hamid Akbari Javar

**Affiliations:** ^1^Department of Pharmaceutics, Faculty of Pharmacy, Hamadan University of Medical Sciences, Hamadan, Iran.; ^2^Department of Pharmaceutics, Faculty of Pharmacy, Tehran University of Medical Sciences, Tehran, Iran.

**Keywords:** Propolis, Solid lipid nanoparticles (SLN), Drug delivery, Solvent emulsification-evaporation method, Box-Behnken design

## Abstract

***Purpose:*** Propolis is a resinous material obtained by honeybees with many biological and pharmacological properties which can be used for treatment of various diseases. Current study aims to formulate and characterize propolis-loaded solid lipid nanoparticles (SLNs) carrier system.

***Methods:*** The prepared SLNs, composed of glyceryl monostearate (GMS), Soy lecithin, Tween 80 and polyethylene glycol 400 (PEG 400), were fabricated employing solvent emulsification-evaporation technique. In addition, the impact of several variables including concentration ratios of GMS/Soy lecithin and PEG 400/Tween 80 along with emulsification time were evaluated on the size, polydispersity index (PDI) and zeta potential of particles. SLN formulations were optimized using Box-Behnken design. The particles were freeze dried and morphologically studied by scanning electron microscopy (SEM). The in-vitro release profile of propolis entrapped in the optimized nanoparticles was investigated.

***Results:*** The mean particle size, PDI, zeta potential, entrapment efficiency (EE) and loading efficiency (LE) of optimized propolis-loaded SLNs were found to be 122.6±22.36 nm, 0.28±0.06, -26.18±3.3 mV, 73.57±0.86% and 3.29±0.27%, respectively. SEM images exhibited nanoparticles to be non-aggregated and in spherical shape. The in-vitro release study showed prolonged release of propolis from nanoparticles.

***Conclusion:*** The results implied that the proposed way of SLN preparation could be considered as a proper method for production of propolis loaded colloidal carrier system.

## Introduction

Natural products are considered to be potential sources of various pharmaceutical compounds. Honeybees are capable of making propolis as a natural resinous product by combining resins gathered from the fissures of the tree bark and leaf buds with their waxes and salivary secretions.^1^ Propolis has been utilized broadly in traditional medicine throughout the history. Over the last decades, several researches have been carried out on identification of the composition, medical applications and biological properties of propolis.^2^ It is employed in cosmeceuticals, either in combination with other natural products or in pure form, and also as a constituent of nutritious foods.^3^ Propolis is a lipophilic material that is hard and breakable in cold temperatures, but by increasing temperature, it converts to a soft, flexible and very adhesive substance.^4^ This natural product possesses many biological and pharmacological properties; for instance, immunomodulatory,^5,6^ anti-tumor,^7,8^ anti-inflammatory,^9^ anti-oxidant,^10-12^ anti-bacterial,^13,14^ anti-viral,^15,16^ anti-fungal,^17,18^ and anti-parasite^19,20^ effects. Beside its merits regarding healing and treatment of wounds, burns and ulcers, propolis is effectively used for the treatment of dermatological, laryngological, and gynecological problems and dental diseases.^21^ According to previous studies, propolis flavonoids are responsible for the majority of these biological and pharmacological properties.^3^ Flavonoids such as, pinocembrin and galangin have been discovered as the major compounds which are responsible for the anti-bacterial activity of propolis.^22^ Moreover, galangin exhibited antioxidant, anti-inflammatory and anti-fungal activities.^23,24^

Solid lipid nanoparticles (SLNs), presented in 1991, have garnered more attention over the past few years. They are believed to be a kind of substitute carrier system for other colloidal nanoparticles like emulsions, liposomes and biodegradable polymer nanoparticles.^25,26^ SLNs are submicron carrier systems composed of a solid lipid/s core coated with surfactants. SLNs particle size varies from 50 to 1000 nm and they are solid at body temperature and room conditions.^27,28^ Noticeable privileges of SLNs comprise controlled release behaviour, insignificant skin irritation, and protection of the loaded active compounds from environmental degradation.^29^ As SLNs consist of physiologically compatible, non-poisonous and non-irritative lipids, they are suggested as an appropriate candidate for administration on inflamed and damaged skin. Furthermore, the suitable contact of SLNs with stratum corneum, which is due to their small size, increases penetration of the quantity of active ingredients into the mucosa or skin. In account of their solid lipid matrix, these nano-carriers can also exhibit a sustained and controlled release of entrapped compounds. It has also been reported that after topical usages, occlusive attributes of SLNs lead to reduction of water loss through the transdermal epithelium and facilitate penetration of the active ingredient through the stratum corneum.^29^

The aim of the present study was to prepare propolis-loaded SLN intended for topical delivery and to optimize the formulation of the nanoparticles employing the Box-Behnken design response surface methodology (BBD-RSM). The physicochemical characteristics including particle size, polydispersity index (PDI), zeta potential, entrapment efficiency (EE), drug loading (LD), morphology, and release behavior of propolis-loaded SLN were investigated.

## Materials and Method

### Materials

Raw propolis samples were collected from a commercial beekeeper, in Tehran (Iran). The collected propolis was stored in a dry place at 4°C for further studies. Galangin and cellulose membrane dialyzing tube (molecular weight cut-off 12000 Da) were procured from Sigma-Aldrich (St. Louis, USA). Glyceryl monostearate (GMS) was obtained from Gattefosse (Gennevilliers, France). Tween 80, Soy lecithin and polyethylene glycol 400 (PEG 400) were acquired from Samchun (Seoul, Korea). Aluminum chloride, dichloromethane, and ethanol (99.7% v/v) were provided from Merk-Millipore (Darmstadt, Germany). De-ionized double distilled water was used wherever required. All other utilized chemicals and solvents were of analytical grade.

### Methods

#### Extraction of propolis

Ethanolic extract of propolis was prepared according to previous reports, with minor modifications.^30^ Briefly, 2 g of propolis was stirred continuously in 180 mL of ethanol at room temperature for 72 hours and the obtained solution was filtered using a Whatman No. 41 filter paper to separate solid impurities. Samples were kept at 4°C and used within 2 weeks of preparation.

In order to standardize the extract, galangin was used as the reference compound for the assay of total flavones and flavonols. The spectrophotometric assay was performed based on the formation of a complex between the aluminum ion (i.e. Al (III)) and the carbonyl and the hydroxyl groups of total flavones and flavonols in the propolis ethanolic extract.^30^

For performing analysis by colorimetric method using UV-Visible spectrophotometer, a stock standard solution of galangin (100 µg/mL) was prepared by dissolving accurately weighed quantity of galangin in ethanol, and series of working standard solutions were made by adequate dilution of the stock solution with ethanol to supply concentrations of 50 µg/mL, 20 µg/mL, 10 µg/mL, 5 µg/mL, and 1 µg/mL. An aliquot (2 mL) of the test solution, 20 mL ethanol, and 1 mL of aluminum chloride in ethanol (5% w/v) were mixed. After 30 minutes, the absorbance was measured at 425 nm by UV–Vis double beam spectrophotometer (Analytik Jena, SPECORD 210 PLUS, Germany). The obtained data revealed proper linearity with a calculated regression coefficient (R^2^) of 0.9976 in the range of 1 µg/mL to 100 µg/mL and also proper precision and accuracy (data not shown).

#### Preparation of SLNs 

The propolis-loaded SLNs were prepared according to a modiﬁed emulsion/solvent evaporation method.^31^ Soy lecithin (50 mg), ethanolic extract of propolis (2.5 mL) standardized using the previously-mentioned method, and appropriate amounts of GMS were dissolved in 2.5 mL dichloromethane as the organic phase. The aqueous phase (5 mL) was made by dissolving Tween 80 (1% w/v) and appropriate amounts of PEG 400 as surfactants. Subsequently, the solution was heated to the same temperature of the organic phase. Afterwards, the organic phase was added dropwise to the hot aqueous phase, stirred at 1000 rpm using magnetic stirrer (Heidolph®, Germany) for an appropriate period of time designated as emulsification time while the temperature was kept constant at 50°C. Upon evaporation of the organic solvent, nanoparticle dispersions were established. In order to solidify the nanoparticles, the colloidal dispersion was transferred to an ice bath and kept stirred at 1000 rpm for one hour. Finally, opalescent colloidal nano-suspension was formed.

The nanoparticle suspension was centrifuged at 14 000 rpm for 30 minutes at 6°C. Then, the settled down nanoparticles were collected and re-dispersed in double distilled water for further studies, and transparent supernatant was utilized for determination of EE% and loading efficiency (LE%) by indirect method as will be explained below.

#### Characterization of nanoparticles

The particle size and PDI of propolis-loaded SLN formulations were measured by photon correlation spectroscopy using a Nano ZS90 Malvern® (Worcestershire, UK). Their associated zeta potential was also measured by laser doppler anemometry using the same equipment. Measurements were carried at an angle of 90° at 25°C. Each measurement was done in triplicate and was reported as mean ± SD.

In order to assess the EE% and the LE% by the indirect method, the opalescent freshly prepared colloidal SLN was centrifuged and the transparent supernatant was analyzed colorimetrically using UV-VIS spectrophotometer at 425 nm, according to the previously-mentioned method. The EE% and LE% of nanoparticles was calculated as follows:

EE% = [(total drug content –

free drug found in the supernatant) /

total drug content)] * 100 (1)

LE% = [(total drug content –

free drug found in the supernatant) /

weight of nanoparticles] * 100 (2)

#### Experimental design studies 

A Box–Behnken design, including 3 factors, 3 levels and 17 runs, was developed for the optimization of nanoparticles utilizing Design-Expert® software version 7.0.0 (State-Ease Inc., Minneapolis, USA). Independent variables (factors) were defined as concentration ratio of GMS/Soy lecithin (A), concentration ratio of PEG 400/Tween 80 (B), and emulsification time (C). Additionally, particle size (Y_1_), PDI (Y_2_), and zeta potential (Y_3_) of the nanoparticles were designated as dependent variables (responses). Table 1 summarizes the ranges and constraints of independent and dependent variables, respectively. The ranges of independent variables were selected using previously-performed preliminary studies and amounts of Soy lecithin, Tween 80 and solidification time were kept constant as 50 mg, 50 mg and 1 hour, respectively.

**Table 1:** Ranges and constrains of variables

**Independent variables (factors)**		**Levels**		
		**-1**	**0**	**+1**
Numeric factors	GMS/Soy lecithin (A)	0	1	2
	PEG 400/Tween 80 (B)	0	2	4
	Emulsification time (C)	0.5	2.75	5
**Dependent variables (responses)**		**Constraints**		
Y_1_=Particle size (nm)		Minimize		
Y_2_=PDI		Minimize		
Y3=Zeta potential (mV)		-20 >Zeta potential		

According to the suggested experimental design, 17 formulations (including 3 center points) were prepared experimentally in triplicate and characterized (Table 2). The obtained data were fitted to the appropriate models (linear, 2-FI and quadratic) and analyzed by the one-way analysis of variance (ANOVA). The models were explained by polynomial equations, and their related 3-D response surface plots were created by Design-Expert® software. For the purpose of model reduction and better predictability, the step-wise method was applied for the elimination of non-significant parameters.

**Table 2:** Box-Behnken experimental design (n=3)

**Run**	**Independent variables**			**Dependent variables**		
	**Factor1** **A: GMS/** **Soy lecithin**	**Factor2** **B: PEG400/** **Tween 80**	**Factor3** **C: Emulsification time**	**Response1** **Particle size (nm)** **(mean ± SD)**	**Response2** **PDI** **(mean ± SD)**	**Response3** **Zeta potential** **(mV)** **(mean ± SD)**
1	1.00	0.00	5.00	183.55±13.9	0.416±0.06	-28.55 **±**2.45
2	0.00	2.00	5.00	58.3**±**3.52	0.64±0.09	-32.8±0.47
3	2.00	4.00	2.75	281**±**29.93	0.397±0.09	-22.05 **±**3.75
4	1.00	4.00	5.00	135**±**5.76	0.466**±**0.05	-18.65 **±**1.95
5	1.00	2.00	2.75	144±11.23	0.58±0.03	-22.4**±**1.23
6	1.00	2.00	2.75	112**±**19.08	0.309±0.05	-18.5**±**2.35
7	0.00	2.00	0.50	56.65**±**3.37	0.561**±**0.12	-34.85 **±**6.15
8	1.00	2.00	2.75	85.3±8.65	0.291±0.07	-14.5**±**1.75
9	1.00	2.00	2.75	84.4±10.36	0.294±0.11	-18.46 **±**2.12
10	2.00	0.00	2.75	159±39.89	0.461±0.02	-19.5**±**3.56
11	1.00	2.00	2.75	110±6.68	0.309±0.09	-34.8**±**2.58
12	2.00	2.00	5.00	225**±**31.23	0.437**±**0.08	-26.25 **±**1.67
13	0.00	4.00	2.75	51.8±2.4	0.525±0.02	-25.25 **±**3.16
14	2.00	2.00	0.50	104.5±21.85	0.358±0.08	-11.22 **±**3.56
15	1.00	4.00	0.50	113.85±27.08	0.483±0.01	-24.85 **±**2.05
16	0.00	0.00	2.75	60.35±15.61	0.652±0.06	-37.4 **±**5.9
17	1.00	0.00	0.50	118.5±12.02	0.358±0.09	-22.5**±**1.63

#### Optimization and model validation

In order to validate the proposed fitted model and evaluate prediction errors indicating the predictability of the system, the optimized formulation suggested by the software was prepared experimentally in five times and characterized in terms of particle size, PDI, zeta potential (mV), EE%, and LE%.

#### Freeze drying of the nanoparticles

The freshly prepared optimized SLN formulation was centrifuged and the supernatant was separated. Afterwards, the settled down nanoparticles were reconstituted using sucrose (3% w/v, 3 mL) as the cryoprotectant and were lyophilized using freeze dryer (Operon®, South Korea). Previous studies have revealed that di-saccharides, such as sucrose are more efficient cryoprotectants compared to mono-saccharides, such as mannitol, sorbitol and trehalose. Consequently they exhibit higher efficiency of conserving the physicochemical features of nanoparticles during lyophilisation.^32^ The freeze-dried powder was re-suspended in distilled water and the physicochemical characteristics including particle size, PDI and zeta potential were evaluated.

#### Morphological studies

The morphology of the lyophilized nanoparticles was examined by scanning electron microscopy (SEM). The nanoparticles were mounted on aluminum stubs and coated with a thin layer of gold, then examined by SEM (JEOL-JSM-6360 JAPAN).

#### In vitro drug release 

In-vitro release of propolis from nanoparticles was evaluated utilizing dialysis bag diffusion method.^33^ The aqueous nano-particulate dispersion of freeze-dried samples was placed in a dialysis bag (molecular weight cut-off of 12 000 Da) and tied tightly at both ends. The sample was submerged in the receptor compartment filled with 100 mL of phosphate buffer solution (pH 7.4) and was stirred continuously in a shaker incubator at 100 rpm (Heidolph®, Unimax 1010 DT, Germany) while the temperature was maintained at 37±2ºC. The volume of the receiver medium was chosen such that the sink condition be ascertained. In order to prevent evaporation of the dissolution medium, the receptor compartment was covered during stirring. The samples (1 mL each) from the receiver compartment were withdrawn at predetermined time points, and an equal volume of previously heated fresh medium was replaced immediately after each sampling.

The samples were analyzed for propolis concentration by spectrophotometric method at 425 nm as mentioned above and the cumulative percentage of propolis released is represented against time.

#### Statistical analysis

In the present study, all the experiments were performed in triplicate except otherwise stated which in those cases the experiments were performed five times. Box-Behnken design and model fitting was accomplished using Design-Expert® software. The significance level was set as 0.05.

## Results and Discussion

### Preparation and characterization of SLNs 

**Figure 1:**
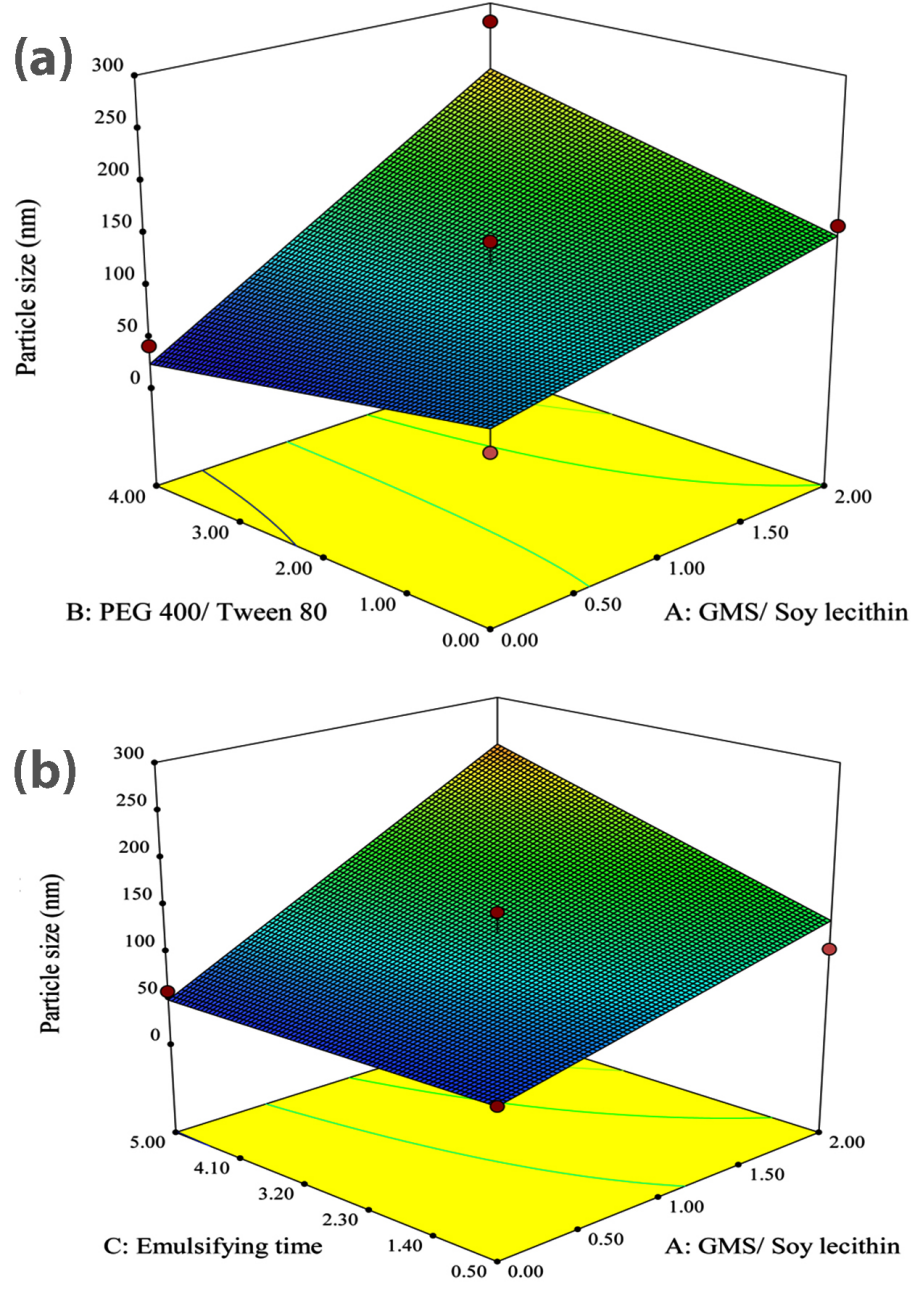
**(a)** 3-D response surface plot of effective parameters on particle size; GMS/ Soy lecithin ratio Vs PEG 400/ Tween 80 ratio. **(b)** 3-D response surface plot of effective parameters on particle size; GMS/ Soy lecithin ratio Vs emulsification time.

**Figure 2:**
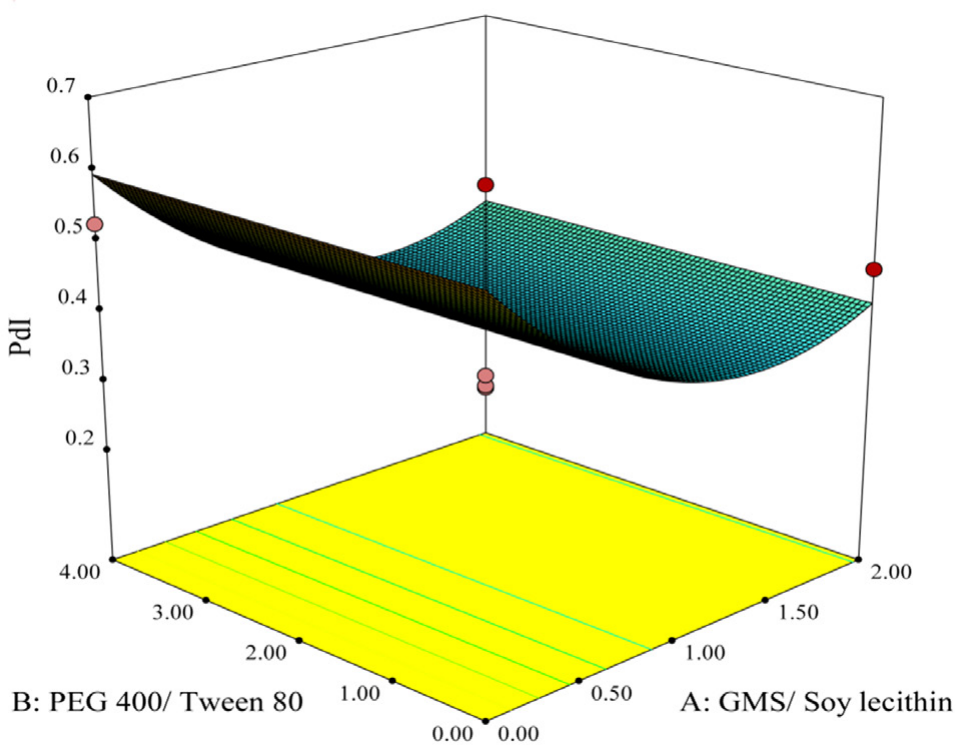
3-D response surface plot of effective parameters on PDI.

**Figure 3:**
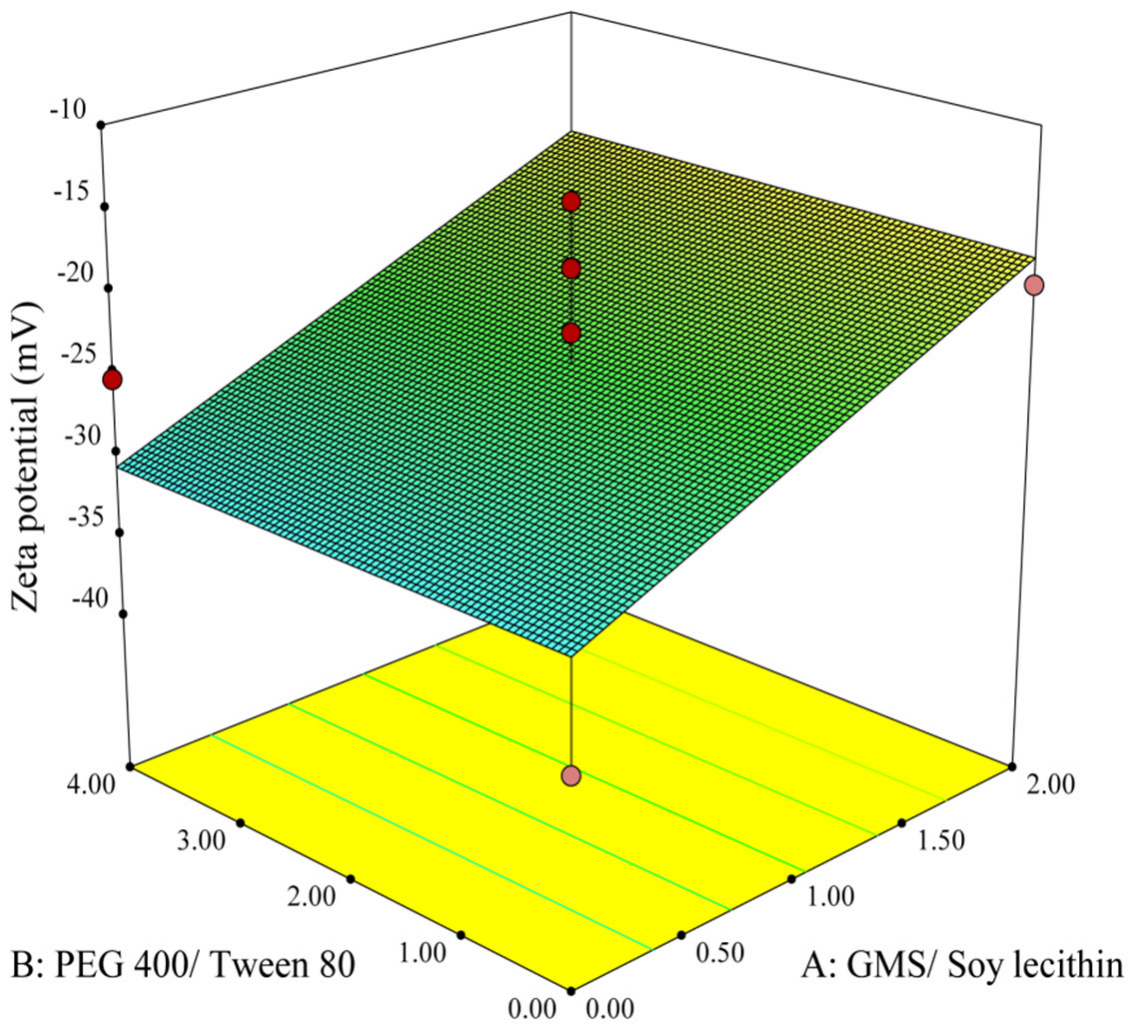
3-D response surface plot of effective parameters on zeta potential.

The data obtained from the experimental preparation of various formulations, which was suggested by the Design-Expert® software, were analyzed and summarized in Table 2. Statistical parameters, such as multiple correlation coefficient, adjusted multiple correlation coefficient, and the predicted residual sum of squares produced by Design-Expert software were used to explain polynomial equations including the main effects and interaction factors. ANOVA provision available in Design-Expert was used for statistical validation of the polynomial equations. Experimental data obtained from software design were employed for determining the variables optimum values based on the desirable constrained criterion, as shown in Table 1. In order to depict the effects of pre-determined factors on the responses including particle size, PDI and zeta potential, the 3-D response surface plots were generated and are demonstrated in Figures 1-3. Observing these 3-D plots, the qualitative and quantitative effects of each factor on the intended responses could be visualized.^34^ The mathematical models were developed to explain the correlation between the factors and the responses. In suggested equations, the negative or positive sign for coefficients indicates a negative or positive effect on the response respectively.^35^

#### Particle size

As presented in Table 2, the particle size varies from 51.8±2.4 nm (formulation No. 13) to 281±29.93 nm (formulation No. 3) in different formulations. The findings were analyzed by ANOVA and utilized to propose the best significant fitted model for the prediction of particle size. The characteristics of the fitted 2-factorial interaction (2-FI) model are summarized in Table 3. This table demonstrates that the model is significant (P < 00.05) whereas lack of fit is non-significant (p>0.05), which implies that the proposed model is adequate for prediction of the response.

**Table 3:** Model characteristics

**Dependent variables (responses)**	***P*** ** value**	**Best fitted model**	**Lack of fit**	**Adeq precision**	**Pred R-squared**	**Adj R-squared**	**R-squared**
Particle size	0.0009	2-FI	Insignificant (P>0.05)	10.936	0.4117	0.6895	0.7672
PDI	0.0042	Quadratic	Insignificant (*P*>0.05)	5.712	0.3805	0.4769	0.5423
Zeta potential	0.0097	Linear	Insignificant (P>0.05)	6.109	0.1912	0.3272	0.3692

Analysis of variance for the fitted model revealed that the main factors of A and C along with binary interaction of A and B have significant effects (*P* < 0.05) on the size of nanoparticles.

The coefficients of significant variables on particle size (Y_1_) are shown in Eq. 3 as follows:

Y_1_= + 121.90 + (69.05*A) + (6.35*B) +

(25.98*C) + (35.14*A.B) (3)

where A, B, and C are the concentration ratio of GMS/ Soy lecithin, the concentration ratio of PEG 400/Tween 80, and the emulsification time, respectively. A.B is defined as a binary interaction effect between A and B, and Y_1_ represents the size of particles. Even though factor B has no significant effect on the size of particles, the binary interaction of A.B has some meaningful influence; the appropriate coefficient of B was involved in the equation due to the hierarchical preservation of the fitted model.

As could be seen in Eq. 3, all the studied factors showed positive effects on the size of particles. The largest coefficient of A indicates the great influence of this variable on the size of nanoparticles.

Figure 1a illustrates the 3D response surface plot showing the alterations of particle size corresponding to changes in either A and B as independent variables. It could be observed from the plot that an increase in the concentration ratio of GMS/Soy lecithin causes an ascent in the mean particle size. These results are perfectly in accordance with the reported data published by Shah et al,^36^ where they showed that the increased amount of GMS led to an increase in particle size. The dependency of lipid nanoparticles size on lipid concentration could be attributed to the tendency of lipid to coalesce at higher concentrations. According to Stoke’s law, this behavior is rooted in the density difference between the internal and external phase.^37,38^ This was also previously reported by Sabapati et al.^39^ Moreover, augmentation of the particle size of SLN due to the increase in the lipid phase concentration could be explained by increasing the viscosity in the lipid-solvent phase that leads to the decrease of solute molecules diffusion rate in the outer phase.^40^ Furthermore, the increase of the particle size could be justified by providing extra space as a result of the increasing amount of lipid, which leads to entrapment of more drug molecules and reduction of the total surface area.^36^

As represented in Figure 1a, the observed increase in the size of particles followed by the increase of GMS is greater in the highest value of PEG 400/Tween 80 concentration ratio (i.e. 4.0) compared to the lowest value of PEG 400/Tween 80 concentration ratio (i.e. 0.0). In this paper, it is assumed that PEG 400 exhibits a lower potential for decreasing the surface tension compared to Tween 80. Therefore, with the increase in the PEG 400/Tween 80 concentration ratio, the reduction in surface tension, which prevents the particle agglomeration, decreases and consequently, the particle size tends to grow. The figure also reveals that in the absence of GMS, the increasing amount of PEG 400 exhibited a non-significant effect on the size of nanoparticles while in the highest concentration ratio of GMS/Soy lecithin (i.e. 2.0), by increasing the concentration ratio of PEG 400/ Tween 80 from 0.0 to 4.0, the size of particles sharply grew.

It is obvious from Figure 1b that the size of particles significantly grew due to the rising emulsification time from 0.5 h to 5.0 h in a manner that the smallest particle size could be obtained after 0.5 hours of emulsification. Rising the emulsification time leads to increasing the amount of lipid incorporated in the core of particles, therefore, the particle size would grow.

#### Polydispersity index 

As shown in Table 2, PDI varies from 0.291±0.07 (formulation No. 8) to 0.652±0.06 (formulation No. 16) in different SLN formulations.

The obtained results analyzed by ANOVA were applied to propose the best significant fitted model for prediction of nanoparticles PDI. The characteristics of the best-fitted model are summarized in Table 3. It could be observed from the table that the proposed quadratic model was significant (*P*< 0.05) while lack of fit was non-significant (P > 00.05), which connotes that the proposed model was appropriate for prediction of the response.

Analysis of the variance for the fitted model revealed that factor A (concentration ratio of GMS/Soy lecithin), considered to be the main factor, along with the square of this parameter have significant effects (*P* < 0.05) on the PDI of nanoparticles. Meanwhile, other main factors or all binary interactions reveal non-significant effects (P > 00.05).

The effect of factor levels on PDI could be described by the following quadratic equation:

Y_2_= +0.039 – (0.091*A) + (0.11* A^2^) (4)

where A represents the concentration ratio of GMS/Soy lecithin and A^2^ is defined as the square of this parameter. Y_2_ is considered as the PDI of the nanoparticles. As could be observed in Eq. 4, factor A has a negative effect on PDI while the square of it affects the PDI positively. In this equation, the major coefficient belongs to A^2^ which indicates the great influence of the square of GMS/Soy lecithin on PDI.

Figure 2 is the 3D response surface plot showing the alterations of PDI associated with the changes in A and B as independent variables. It could be observed from the plot that increasing the concentration ratio of GMS/Soy lecithin from 0.00 to 1.35 leads to PDI reduction to its minimum value, but further rises in the ratio causes a slight increase in this factor.

#### Zeta potential 

According to the experimentally obtained results summarized in Table 2, the value of zeta potential varied from -11.22±3.56 mV (formulation No. 14) to -37.4 ±5.9 mV (formulation No. 16).

The obtained results analyzed by ANOVA and were utilized to propose the best significant fitted model for the prediction of the zeta potential of nanoparticles. The characteristics of the linear fitted model are summarized in Table 3. According to this table, the model was significant (P < 00.05) while lack of fit was non-significant (P > 00.05), which implies that the proposed model was suitable for prediction of the response.

Analysis of the variance for the proposed model revealed that among all main factors, only factor A (concentration ratio of GMS/ Soy lecithin) showed a significant influence on zeta potential of the particles (*P* <0.05) whereas other main factors and their associated binary interactions showed non-significant influences on this parameter (P > 00.05).

The Linear model of zeta potential is shown in Eq. 5 as follows:

Y_3_= - 24.27+ (6.41*A) (5)

where A and Y_3_ are defined as concentration ratio of GMS/Soy lecithin and zeta potential of the particles, respectively. As could be seen in Eq. 5, factor A showed a significant positive effect on the zeta potential.

The 3D response surface plot in Figure 3 illustrates variations of zeta potential corresponding to the changes in A and B as independent variables. The plot shows that an increase in the concentration ratio of GMS/Soy lecithin triggers a sharp rise in the zeta potential of the particles. This finding is in accordance with the study performed by Sahu et al,^40^ which implied that an increasing amount of GMS leads to augmentation of the zeta potential of nanoparticles. Furthermore, in a study done by Wang et al,^41^ it was demonstrated that zeta potential is more related to the type of lipid rather than other factors.

### Optimization and model validation

The optimization of the physicochemical characteristics of SLNs was carried out by statistical analysis of experimentally obtained data. The objective criteria for optimization were defined as minimization of particle size, PDI value, and zeta potential. The optimized and predicted conditions for preparation of SLN are shown in Table 4. As shown in the table, the optimal values for the concentration ratio of GMS/Soy lecithin (A), the concentration ratio of PEG 400/Tween 80 (B), and emulsification time (C) were predicted as 1.35, 0.00 and 0.5 hours, respectively.

**Table 4:** Optimized independent variables and predicted responses

**Optimized independent variables**			**Predicted dependent variables (responses)**			**Desirability**
GMS/Soy lecithin	PEG 400/Tween 80	Emulsification time	Particle size (nm)	PDI	Zeta potential (mV)	0.667
1.35	0.00	0.50	101.564	0.37201	-21.9999	

To determine the model validation and calculate prediction error, the nanoparticles were experimentally prepared and characterized (n = 5). The observed responses and calculated values of the predicated errors are indicated in Table 5. According to the table, the evaluated prediction errors were below 10% for all the factors demonstrating significance, efficiency, and adequacy of the fitted models for prediction of the responses. The diameter of the optimized particles was measured 111.3±19.35, which exhibits a proper occlusive effect on the skin and increases skin penetration of the active ingredient.^42^ The PDI value indicates the homogeneity of particle size distribution in a colloidal dispersion. A small value of PDI indicates narrow particle size distribution in the system whereas large value shows broader distribution.^43^ The experimentally obtained PDI value of 0.34±0.05 demonstrates that the optimized propolis-loaded SLNs exhibit relatively homogenous particle size distribution. Zeta potential is considered as a proper index to determine the stability of colloidal dispersions.^44^ The experimentally obtained zeta potential value of -24.17±3.3 mV indicates a high stability in particles due to the establishment of the strong electrostatic repulsive forces among particles that prevent aggregation upon storage. The negative value of zeta potential could reveal the presence of fatty acid in outer structure of SLN.^45^

**Table 5:** Predicted and observed values for the model validation (n=5)

**Dependent variables (Responses)**							
**Particle size (nm)**		**PDI**		**Zeta potential (mV)**		**EE%**	**LE%**
**Observed response** **(Mean±SD)**	**Prediction error (%)**	**Observed response** **(Mean±SD)**	**Prediction error (%)**	**Observed response** **(Mean±SD)**	**Prediction error (%)**	**Observed response** **(Mean±SD)**	**Observed response** **(Mean±SD)**
111.3±19.35	-9.58	0.34±0.05	8.6	-24.17±3.3	9.86	73.57±0.86	3.29±0.27

### Freeze drying 

Lyophilization is believed to be a suitable method for increasing the chemical and physical stability of products over prolonged periods. In this method, transformation of products into solid-state by removing water from them would prevent the Ostwald ripening phenomenon and avoid hydrolytic reactions.^46^

The particle size, PDI and zeta potential of the optimized formulation following lyophilization of nanoparticles were measured and compared with physicochemical characteristics of particles ahead of lyophilization (n=5). As shown in Table 6, although freeze drying caused a significant increase in the size of particles and PDI (*P*>0.05), the changes in zeta potential were not significant (*P* <0.05).

**Table 6:** Physico-chemical properties of propolis nanoparticles; before and after lyophilization (n=3)

**Physico-chemical characteristics**	**Before lyophilization**	**After lyophilization**
Particle size (nm) (Mean±SD)	111.3±19.35	171.1±18.97
PDI (Mean±SD)	0.34±0.05	0.36±0.05
Zeta potential (mV) (Mean±SD)	-24.17±3.3	-26.87±2.04

### Morphological studies

**Figure 4:**
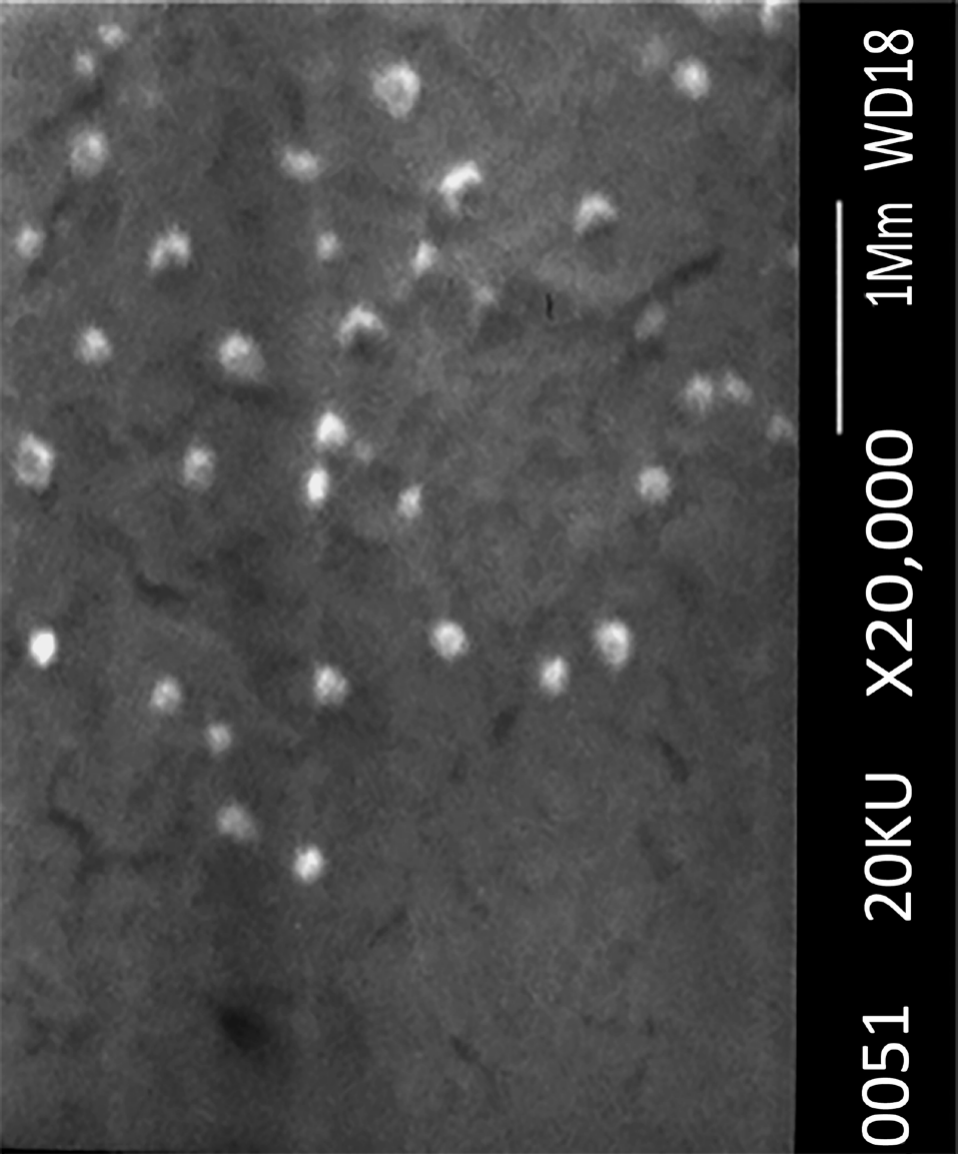
SEM image of optimized nanoparticles.

The morphology of the optimized nanoparticles was studied by SEM. Figure 4 illustrates SEM image of the prepared optimized propolis-SLN revealing that the lyophilized particles were spherical, they remained not aggregated and their sizes were in good accordance with the data obtained by dynamic laser scattering.

### In vitro release studies

**Figure 5:**
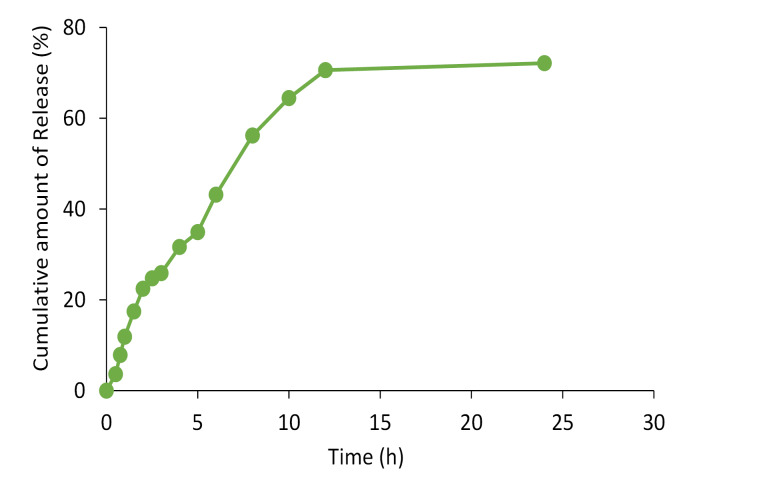
The cumulative in-vitro release profile of propolis from nanoparticles (n=3).

The release of propolis entrapped into nanoparticles was investigated using a dialysis bag. The release profile of propolis from prepared SLN over 24 hours is represented in Figure 5. The initial release of propolis from the nanoparticles could be described by considering desorption of propolis from the outer surface of the SLNs. Prolonged-release of the entrapped compound in the later phase is attributable to the slow diffusion of propolis from the solid lipid matrix. This is in agreement with the studied done by Sood et al.^47^ The observed slow release of homogeneously dispersed propolis in the lipid matrix of the SLN preparation is in accordance with the previous studies.^48,49^

## Conclusion

In the present paper, the SLNs containing propolis were successfully prepared employing the modified emulsion-solvent evaporation method. The influence of independent variables on the particle size, PDI and zeta potential was evaluated by a Box–Behnken design. Subsequently, the formulation parameters were statistically optimized and successfully prepared. The physicochemical characteristics of the designed formulation revealed that SLNs could be regarded as an appropriate colloidal carrier system, in view of the fact that they showed small particle size with a spherical shape, narrow size distribution, suitable zeta potential, and other desirable physicochemical properties, including high values for EE% and LE%. The nanoparticles exhibited a prolonged release of propolis. Additional studies would be of necessity for the evaluation of efficiency after topical application.

## Ethical Issues

Not applicable.

## Conflict of Interest

The authors declare no conflict of interests.

## Acknowledgments

This study was performed as the Pharm.D dissertation of Sahar Taherzadeh (Pharm.D candidate, Faculty of Pharmacy, Hamadan University of Medical Sciences) and was made possible by financial supports from deputy of research and technology, Hamadan university of Medical Sciences, Hamadan, Iran under a grant [No. 9503251530].
